# Antibacterial Films Based on PVA and PVA–Chitosan Modified with Poly(Hexamethylene Guanidine)

**DOI:** 10.3390/polym11122093

**Published:** 2019-12-13

**Authors:** Ewa Olewnik-Kruszkowska, Magdalena Gierszewska, Ewelina Jakubowska, Iwona Tarach, Vladimir Sedlarik, Martina Pummerova

**Affiliations:** 1 Faculty of Chemistry, Nicolaus Copernicus University in Toruń, 7 Gagarina street, 87-100 Toruń, Poland; ewelina@doktorant.umk.pl (E.J.); tarach@doktorant.umk.pl (I.T.); 2 Centre of Polymer Systems, University Institute, Tomas Bata University in Zlin, tr. Tomase Bati 5678, 760 01 Zlin, Czech Republic; sedlarik@utb.cz (V.S.); pummerova@utb.cz (M.P.)

**Keywords:** poly(vinyl alcohol), chitosan, antibacterial properties, poly(hexamethylene guanidine), polymeric films, surface properties

## Abstract

In this study, thin, polymeric films consisting of poly(vinyl alcohol) (PVA) and chitosan (Ch) with the addition of poly(hexamethylene guanidine) (PHMG) were successfully prepared. The obtained materials were analyzed to determine their physicochemical and biocidal properties. In order to confirm the structure of PHMG, nuclear magnetic resonance spectroscopy (^1^H NMR) was applied, while in the case of the obtained films, attenuated total reflectance infrared spectroscopy with Fourier transform (FTIR-ATR) was used. The surface morphology of the polymer films was evaluated based on atomic force microscopy. Furthermore, the mechanical properties, color changes, and thermal stability of the obtained materials were determined. Microbiological tests were performed to evaluate the biocidal properties of the new materials with and without the addition of PHMG. These analyses confirmed the biocidal potential of films modified by PHMG and allowed for comparisons of their physicochemical properties with the properties of native films. In summary, films consisting of PVA and PHMG displayed higher antimicrobial potentials against Gram-positive (*Staphylococcus aureus*) and Gram-negative (*Escherichia coli*) bacteria in comparison to PVA:Ch-based films with the addition of PHMG.

## 1. Introduction

The development of materials with antibacterial properties belongs to a research area that is constantly and intensively expanding. Materials with the potential to inhibit the proliferation of bacteria pose a huge potential for applications in various industry sectors such as packaging production. Different methods of polymer modification allow for the improvement of hygiene or sterility, maintaining the desired quality of products and providing protection against pathogenic microorganisms [1,2,3]. Currently, scientists’ interest is increasingly focusing on using biodegradable polymers for this purpose [1,2,4,5,6]. Poly(vinyl alcohol) (PVA) and chitosan (Ch) [7,8,9,10,11,12,13] are the best known environmentally friendly polymers. PVA is a polymer that displays very useful functional characteristics, i.e., a high mechanical strength, photostability, and high gas barrier properties (especially in relation to oxygen) [4,13]. PVA belongs to a small group of polymers that are not obtained in the direct polymerization of vinyl alcohol monomer. It is well known that vinyl alcohol is an unstable compound that spontaneously rearranges into acetaldehyde. For this reason, poly(vinyl alcohol) is obtained through a hydrolysis reaction of poly(vinyl acetate) [4]. One of the most significant features of PVA is its hydrophilicity, which is the result of the presence of numerous hydroxyl groups in the main chain of the macromolecule [12]. Moreover, it should be mentioned that poly(vinyl alcohol) is characterized by unique properties, such as excellent film forming properties, high crystallinity, nontoxicity, and a remarkable chemical resistance. Because of the above-mentioned features, PVA is of considerable interest to researchers. Publications in this field have included a large number of papers devoted to the formation of PVA-based mixtures with other polymers such as chitosan, agar, and poly(ethylene glycol) [14,15], as well as to PVA modification by means of different additives [4,10,16]. Chitosan is obtained as a result of the deacetylation of chitin. It is commonly acknowledged as a nontoxic, natural, biodegradable and biofunctional material; therefore, it is considered to be safe for human organisms. Moreover, some authors have claimed that chitosan has antifungal and antibacterial properties [12,13,17,18,19]. The antibacterial properties of chitosan are related to its polycationic character [12,18,20]. Protonated functional chitosan groups interact with negatively charged cell membranes of microorganisms, causing damage and, eventually, eradication. However, these properties are strongly dependent on the basic biopolymer features such as the molecular weight or degree of deacetylation, as well as the external conditions in which the material is used. Qin et al. [19] stated that the antibacterial potential of chitosan dissolved in acidic media can be attributed to the presence of -NH_3_^+^ functionals. They also found that chitosan itself (degree od deacetylation (DDA) circa 50%, low molecular weight, and water soluble) had no antibacterial effect on *Escherichia coli* and *Staphylococcus aureus* in a non-acidic environment. Therefore, additional antimicrobial agents (e.g., cinnamon oil, rosemary extract, α-tocopherol, and tea tree essential oil) are frequently being used to synergistically increase the biopolymer’s ability to combat microorganisms on the surface of particular materials [21,22,23].

Both poly(vinyl alcohol) and chitosan, as well as materials based on them, have found many applications in medicine, pharmaceuticals, and materials that come into contact with food. As mentioned above, this is in particular due to their biocompatibility, biodegradability, and low or even complete lack of toxicity [4,12,17,18]. The ways in which these polymers are modified mostly contribute to the improvement of the properties of the materials [12]. In the case of PVA and chitosan, their good miscibility is the result of the hydrogen bonds formed between their functional groups. Therefore, blending PVA and Ch contributes to receiving homogeneous materials with antimicrobial properties and better mechanical properties than chitosan [4,9].

In addition to chitosan [9], the most common antibacterial additives that are used in the modification of PVA’s potential to inhibit the growth of microorganisms include silver nanoparticles [16], ZnO beads [10], plant extracts [24], nanocellulose [25], N-halamine [8], and poly(hexamethylene guanidine) (PHMG) [4]. PHMG is an antibacterial agent that is used in the range of antimicrobial polymeric materials [26,27]. PHMG is obtained during the polycondensation of guanidine with hexamethylenediamine [28]. The modification conditions necessary to obtain poly(hexamethylene guanidine) with a specific structure and properties require careful adjustment [26,27,28,29]. PHMG impedes the proliferation of bacteria, fungi and viruses [4,30]. The above-mentioned properties allow for the application of these compounds as a disinfecting component [27]. The biocidal properties of PHMG are frequently described as being the result of PHMG molecules’ electrostatic destructive interaction with the anionic surface of bacterial cell membranes [30]. In relation to the research by Walczak [27] and Richert [26], it has been established that the incorporation of a biologically active agent (in the form derivatives of PHMG) into a polylactide provides an effective decrease of the proliferation of microorganisms and the slight aggravation of the mechanical properties of the obtained composites. In turn, in Chen’s work [4], the formation of the secondary bonds between poly(hexamethylene guanidine) and poly(vinyl alcohol) was found to cause an improvement in the mechanical properties of PVA-PHMG materials, in comparison with neat PVA. Moreover, the incorporated active agent, once introduced, is permanently present in the polymer matrix and provides antibacterial properties [4].

Due to chitosan’s capacity to impede bacteria growth through ionic interactions with negatively charged cell walls, numerous studies have focused on PVA modification with this biopolymer as an antimicrobial additive. Moreover, it has to be mentioned that PVA belongs to the group of thermoplastics, like most other polymers used in the packaging industry [8]. Thus, poly(vinyl alcohol) modifications created through blending with Ch and the incorporation of an additional antibacterial agent into the matrix are likely to result in the formation of new materials with better antimicrobial properties. To improve this property in non-acidic media, the addition of biocidal agents should be considered. Therefore, the purpose of this study was to obtain new materials consisting of PVA and PVA–Ch blends with or without the addition of PHMG as the active agent, as well as to characterize their antibacterial and physicochemical properties. There have been no papers devoted to the analysis of the effect of both chitosan and PHMG (two antibacterial components) on the physicochemical and biological properties of PVA-based materials. The obtained results allowed for an evaluation of a likely increase or decrease in the antibacterial effect of PVA-based materials modified with both of the biologically active compounds.

## 2. Materials and Methods

### 2.1. Materials

**Figure 1:**
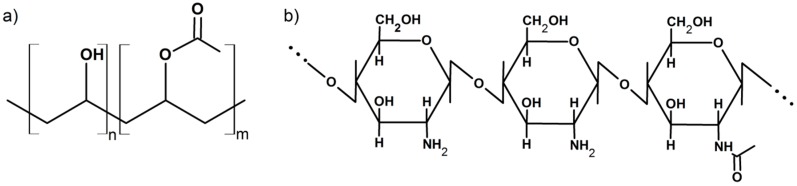
Chemical structure of (**a**) partially hydrolyzed poly(vinyl alcohol) (PVA) and (**b**) chitosan.

Poly(vinyl alcohol) (PVA) with average molecular weight of 31–50 kg·mol^−1^ and degree of hydrolysis (*DH*) equal to 87–89 mol% was delivered by the Sigma-Aldrich company (Germany) (Figure 1a). Commercially available chitosan (Ch) from crab shells with a degree of deacetylation (*DDA*) determined by potentiometric titration equal to 72.25 ± 0.77% and a viscosity average molecular weight (*M_v_*) of the chitosan solution equal to 148 ± 26 kg·mol^−1^ was purchased from BioLog Heppe GmbH (Landsberg, Germany) (Figure 1b). Formic and acetic acids were purchased from Avantor Performance Materials Poland S.A. (Gliwice, Poland). All of the used chemicals were of high purity. Deionized water was used throughout the entire study.

For the poly(hexamethylene guanidine) synthesis, guanidine chloride (CH_5_N_3_, M_w_ = 95.53 g∙mol^−1^) and hexamethylenediamine (C_6_H_16_N_2_, M_w_ = 116.20 g∙mol^−1^) were purchased from Sigma-Aldrich (Germany). A hydrochloric acid (HCl, 35–38% cz.d.a., purchased from Avantor Performance Materials Poland S.A., Gliwice, Poland) was used for the neutralization of the polycondensation byproduct.

The media required for antimicrobial testing (nutrient broth, Mueller–Hinton agar, plate count agar) were bought from HiMedia Laboratories (Mumbai, India). The bacterial strains *Staphylococcus aureus* (CCM 4516) and *Escherichia coli* (CCM 4517) were obtained from the Czech Collection of Microorganisms, Masaryk University in Brno, Czech Republic.

### 2.2. Synthesis of Poly(Hexamethylene Guanidine)

**Figure 2:**
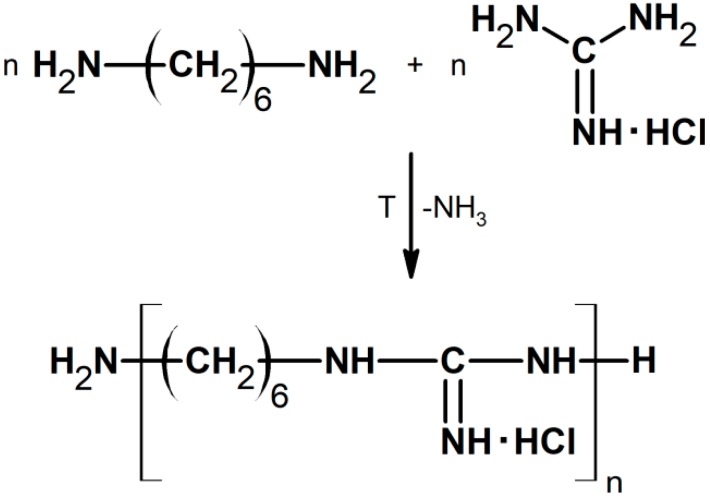
Scheme of poly(hexamethylene guanidine) (PHMG) reaction synthesis.

In order to obtain PHMG equimolar amounts of substrates, 1,6-hexamethylenediamine and guanidine hydrochloride were weighed and introduced into a 100 mL flask. The reaction was carried out at 100 °C for 30 min, and then the temperature was raised by 20 °C. This operation was repeated every 30 min until the temperature reached 180 °C, which was maintained throughout the entire duration of the process (3 h). At the end of the reaction (Figure 2), PHMG was obtained as a colorless liquid with a high viscosity that solidified after cooling. The ammonia, being a byproduct during the PHMG synthesis, was introduced and neutralized in a beaker containing an HCl solution. The structure of the obtained materials was confirmed by means of the ^1^H NMR technique.

### 2.3. The Preparation of Polymeric Films

#### 2.3.1. PVA and Chitosan Solutions

To prepare the 5 wt.% PVA solution, a predetermined mass of PVA powder was dispersed and then fully dissolved in distilled water at 80 °C. The 1 wt.% chitosan (Ch) solution was prepared by dissolving a predetermined mass of chitosan flakes in 2% (w/v) acetic acid. The obtained mixture was filtered to remove insoluble impurities.

#### 2.3.2. The Formation of Films

All polymeric films were obtained by using the casting and the solvent evaporation technique. Both the pure PVA solution and the PVA and Ch mixture (Ch:PVA = 1:1 mass ratio) were cast on a clean polystyrene (PS) plates, dried for 48 h at 37 °C, and subsequently placed in vacuum for 24 h at the same temperature. The ratio between the solution volume and the PS plate surface was adjusted to reach a 0.1 mm film thickness. As a result, pristine polymeric films PVA-0 and PVA:Ch-0 were obtained (Table 1).

Films containing PHMG were obtained according to the above-described procedure with some modifications. The PHMG sample weight, being 0.5 or 1 wt.% of the PVA or PVA:Ch polymer mass, was dissolved in a low amount of water and mixed with the PVA solution before (i) casting (neat PVA films) or (ii) adding the Ch solution and casting (two component PVA:Ch films). The obtained mixtures containing PHMG were cast and dried as PVA-0 and PVA:Ch-0.

All obtained films, whose compositions are presented in Table 1, were stored in a desiccator over P_2_O_5_ at ambient temperatures for further testing.

### 2.4. Methods of Analysis

#### 2.4.1. ^1^H-NMR Analysis

In order to confirm the structure of the obtained PHMG, a sample analysis was carried out by using nuclear magnetic resonance (^1^H NMR) spectroscopy. The sample was dissolved in heavy water (D_2_O). The NMR spectrum was recorded on a BRUKER spectrometer (Rheinstetten, Germany) at 700 MHz.

#### 2.4.2. Fourier Transform Infrared Spectroscopy (FTIR)

The Fourier transform infrared–attenuated total reflectance (FTIR-ATR) spectra of all polymeric films were recorded on a Nicolet iS10 (Thermo Fisher Scientific, Waltham, MA, USA) equipped in an ATR device (attenuated total reflectance) with a zinc–selenide crystal in the frequency range of 500–4000 cm^−1^. The spectra of PHMG in KBr disc form were recorded with this same device working in transmittance mode. All spectra were recorded at a resolution of 4 cm^−1^, scanned 64 times, and analyzed using the OMNIC 7.0 software (Thermo Fisher Scientific, Waltham, MA, USA).

#### 2.4.3. Atomic Force Microscopy (AFM)

An atomic force microscope (AFM) (NanoScope MultiMode, Veeco Metrology, Inc., Santa Barbara, CA, USA) was used to analyze the surface roughness and surface topography of all polymeric films. Analyses were performed in air, at ambient temperature, and in AFM tapping mode. Films were cut into 1 × 1 cm pieces prior to measurement. Eventually, two roughness parameters were determined by using a scan area of 5 × 5 μm: (i) the roughness average (R_a_), which is defined as “an average of the absolute values of the surface height deviations measured from the mean plane,” and (ii) the root mean square (R_q_), which is defined as “the root mean square average of height deviations taken from the mean data plane.” The R_q_ and R_a_ values were the average values of three independent scans.

#### 2.4.4. Contact Angle Measurements

Contact angle measurements were used to evaluate the possible interactions between films and bacteria cells that influenced bacterial adhesion and, thus, the biofilm formation process. The surface hydrophilicity of all polymeric films was determined by using two various testing liquids: a non-polar diiodomethane (DIM) and bipolar glycerin (G), according to the standardized method under the ISO 8296:2003 International Standard 2004 using the G10/DSA10 Drop Shape Analyzer (KRÜSS Inc., Germany) at approximately 22 °C and at 50% relative humidity. The results of five independent measurements for each film and each testing liquid were averaged and recalculated with the Owens, Wendt, Rabel and Kaelble (OWRK) method [31] into total surface free energy (SFE, $\gamma_{S}$) and their polar ($\gamma_{S}^{p}$) and dispersive ($\gamma_{S}^{d}$) components.

#### 2.4.5. Color Measurement

The differences in the color of the obtained polymer film, with and without the addition of PHMG, were analyzed by means of a MICRO-COLOR II LCM 6 (Dr Lange) colorimeter. The CIE L*a*b* system, which is based on the opponent theory of color vision, was applied in order to established differences in color between a pair of materials. The color difference (Δ*E*) was calculated by using Equation (1):

(1) $$\Delta E=\sqrt{{\left(L-L^{*}\right)}^{2}+{\left(a-a^{*}\right)}^{2}+{\left(b-b^{*}\right)}^{2}},$$

where *L* is the lightness component and *a* and *b* represent chromatic parameters. In the case of the “*a*” parameter, the colors ranged from green (−*a*) to red (+*a*), and the “*b*” parameter changed from blue (−*b*) to yellow (+*b*). L*, a*, and b* are the standard color values in a material that is used a control sample (in this case, it was a PVA film), while *L*, *a* and *b* are the values of films with the addition of chitosan and/or PHMG. In this study, five measurements were performed for each of the studied materials, then the values were averaged.

#### 2.4.6. Thermogravimetric Analysis

Thermogravimetric (TG) analyses of the PVA and the PVA-based materials were carried out on simultaneous DTA-TGA thermal analyser type SDT 2960 (TA Instruments, London, UK). All measurements were performed at a heating rate of 10 °C·min^−1^ under air flow from room temperature to 600 °C.

#### 2.4.7. Mechanical Properties

The mechanical properties of PVA, poly(vinyl alcohol)-chitosan films, and materials with the addition of PHMG were analyzed by means of the 27025 TIRA machine test, which is dedicated to mechanical testing. The crosshead speed was 20 mm·min^−1^ with an applied 100 N force. The measurements were performed according to the PN-EN ISO 527-1, -3 standard. In the case of each type of studied materials, at least seven samples were analyzed, and, based on the obtained data, the standard deviation was calculated. The linear region of extension curves allowed us to establish the Young’s modulus (E).

#### 2.4.8. Disk Diffusion Method

The antibacterial properties of the obtained PVA films were evaluated by means of the disk diffusion method [16]. In order to perform the biocidal test, *Staphylococcus aureus* (CCM 4516) and *Escherichia coli* (CCM 4517) bacteria representative species were used in their suspension form with a bacterial concentration ranging between 10^6^ and 10^7^ CFU/mL. Two samples (with dimensions of 1 by 1 cm) of each material type were placed onto one Petri dish with Mueller–Hinton agar (pH 7.4 at 25 °C) inoculated with bacteria suspensions. Plates were prepared in duplicate and incubated at 35 °C for 18–24 h. After the incubation, the width of the inhibition zone in the case of each sample was measured.

#### 2.4.9. Dilution and Pour Plate Culture Method

The dilution and pour plate culture method was the second method used to assess the antimicrobial properties of the PVA-based materials. A 0.1 g sample in a tube with a 9 mL 1/500 nutrient broth (pH 7.4 at 25 °C) was inoculated with 1 mL of bacteria suspension. The tube content was subsequently homogenized and incubated at 35 °C for 24 h. The biocidal test was carried out thrice in the case of each sample type by using two representative bacteria species in suspensions characterized by the same concentration—as was also applied in the previous procedure. After homogenization, tubes containing the same types of materials were combined with each other in order to obtain an average sample; afterwards, they were homogenized again. Subsequently, a series of 10-fold dilutions of samples was performed. One milliliter of each dilution and 20 mL of PCA (plate count agar)-containing neutralizers (Tween 5 g/L and Lecithin 0.7 g/L) were poured into a Petri dish and mixed well. All duplicate plates were incubated at 35 °C for 48 h. After the defined time period, the number of bacteria colonies in the Petri dishes was determined.

The antibacterial activity (R) was calculated using Equation (2):

(2) $$R=log\;N_{blank}\;-log\;N_{sample},$$

where $log\;N_{blank}$ and $log\;N_{sample}$ are the logarithms of viable bacteria number in the reference and modified sample, respectively. Additionally, in order to compare results with the ones obtained by other researchers, the inhibition rate of cell growth was calculated according to the formula presented by Chen et al. [4].

## 3. Results and Discussions

### 3.1. Analysis of PHMG Structure by Means of NMR Technique

**Figure 3:**
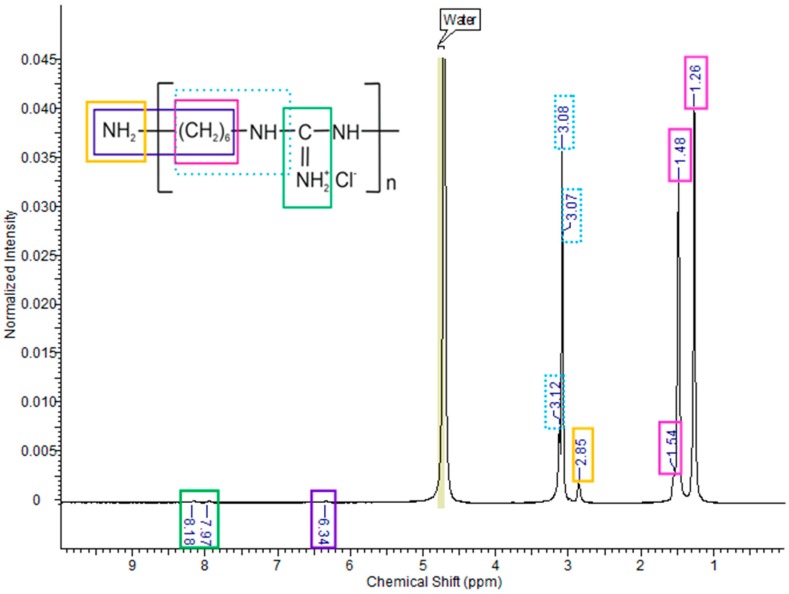
^1^H-NMR spectrum of the PHMG.

In order to establish the structure of the obtained poly(hexamethylene guanidine), an ^1^H-NMR analysis was performed. The chemical shifts of the particular protons and groups of atoms originating from PHMG are presented in Figure 3. The signal with the highest intensity at 4.71 ppm could be ascribed to the solvent used in the process, i.e., heavy water. Based on the available literature [30,32], subsequent signals were assigned to the following groups of atoms: 1.26–1.54 ppm: -CH_2_-; 2.85 ppm: -NH_2_; 3.07–3.12 ppm: -CH_2_–NH-; 6.34 ppm: NH_2_–CH_2_; 7.97–8.16 ppm: C=NH_2_^+^. When describing the location and intensity of the protons present in the structure of PHMG, we have to bear in mind that protons belonging to CH_2_ are difficult to distinguish and that NH and CH signals can overlap each other [32].

**Figure 4:**
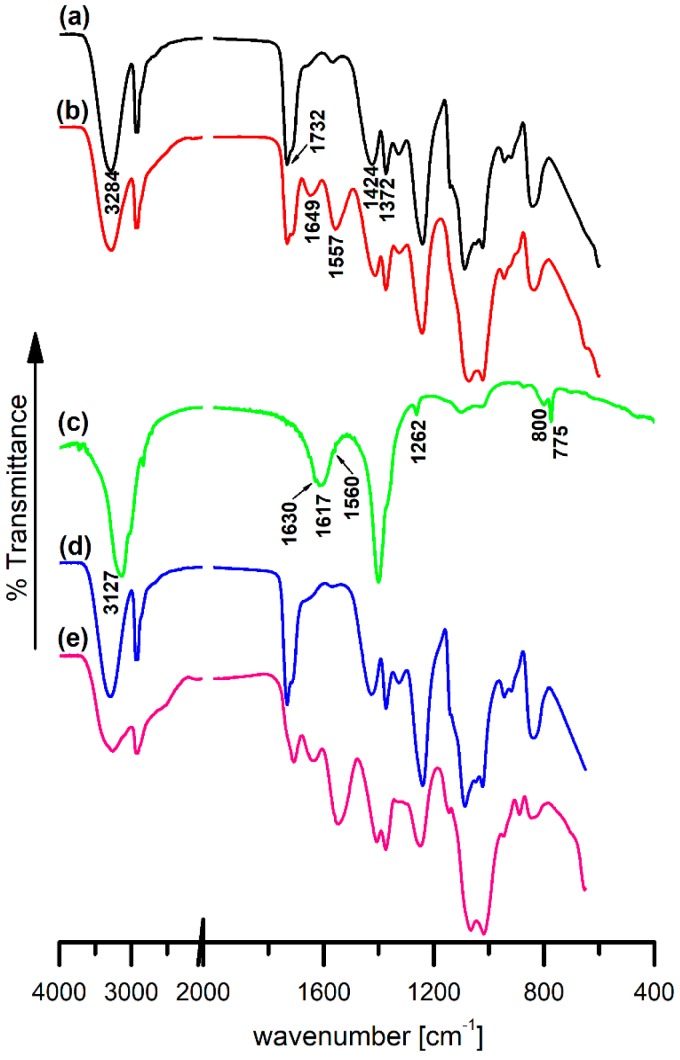
FTIR-ATR (Fourier transform infrared–attenuated total reflectance) spectra of PVA-0 (**a**), PVA:Ch-0 (**b**), PVA-1.0 (**d**), PVA:Ch-1.0 (**e**) films and the FTIR spectrum of PHMG powder (**c**).

In order to confirm that the PHMG polymer was indeed obtained, the FTIR technique was applied. The FTIR spectrum of PHMG is presented in Figure 4c, and the characteristic bands are described in Section 3.2 dedicated to the FTIR analysis of all studied materials. Taking into account the signals present in the NMR spectrum, as well as the bands observed during the FTIR analysis, it was established that both techniques confirmed that poly(hexamethylene guanidine) was actually obtained in the process.

### 3.2. Fourier Transform Infrared Spectroscopy

FTIR spectroscopy is an instrumental method that allows one to study changes in the structural and chemical properties of materials and substances obtained as a result of chemical modifications applied to them. The FTIR spectra of pristine PVA-0- and PVA:Ch-0-based films, as well as the PHMG and PHMG-containing PVA and PVA:Ch films, are presented in Figure 4. In the spectrum of the neat PVA-0 film (Figure 4a), a broad band in the 3000–3600 cm^−1^ region with a maximum value of 3284 cm^−1^ is visible and can be ascribed to O–H stretching vibrations and intermolecular hydrogen bonding. Two bands with maximum values of 2938 and 2909 cm^−1^ corresponded to asymmetric and symmetric stretching C–H vibrations in -CH_2_-. Additionally, other bands corresponded to the presence of -CH_2_- groups in the polymeric chain: bending C–H vibrations at 1424 cm^−1^ and wagging C–H vibrations at 1372 cm^−1^ [33]. The band at 1732, which can be attributed to C=O vibrations, confirmed the presence of residual acetate functional groups in the partially hydrolyzed PVA [34,35,36]. All other vibrations at 1326 (O–H bending vibration), 1240 (C–H wagging vibrations in acetate residue) and 1087 cm^−1^ (O–C–O stretching vibrations) were also characteristic of the PVA structure [34,35,36].

A comparison of FTIR spectra of the pristine one-component PVA-0 and two-component PVA:Ch-0 films (Figure 4a,b) revealed certain differences in the regions that corresponded to typical bands of chitosan. PVA and Ch possess large number of functional groups that are involved in the formation of hydrogen bonds, namely -OH in PVA and both -OH and -NH_2_ in Ch, respectively [33,34,35,36,37,38]. Mixing PVA with Ch resulted in the breaking of existing H-bonds, while new ones joining PVA and Ch could be formed. Therefore, certain changes in the intensity of bands in the 3500–3150 cm^−1^ range could be observed. Moreover, there were also visible new intensive bands at 1649 and 1557 cm^−1^ in the PVA:Ch-0 spectra (Figure 4b). These bands could be assigned to the vibrations of bonds present in the chitosan structure: C=O stretching vibrations in the amide I group and N–H bending vibrations in the amide II group [37,38].

In the FTIR spectrum of PHMG (Figure 4c), the peaks characteristic of the guanidine group could be observed: at 3127 (N–H stretching), 1630 (C=N stretching), 1617 (N–H bending) and 1262 cm^−1^ (C–N stretching). There was also a small band at 1560 cm^−1^ visible on the shoulder of the N–H bending vibration band that could be assigned to the bending vibration of the residual NH_2_^+^ moieties [32,33,39]. The presence of the -NH_2_ primary amine could be also confirmed by the presence of bands at 775 and 800 cm^−1^ that corresponded to N–H wagging vibrations [33].

A comparison of the neat polymeric films’ spectra (Figure 4a,b) with the corresponding spectra of the PHMG-containing films (PVA-1.0 Figure 4d and PVA:Ch-1.0 Figure 4e) (overlapped spectra in Supplementary Materials (Figure S1)) revealed that there was no evident difference between the intensity and the maximum positions of all characteristic bands. As was previously found by Kukharenko et al. [40], the incorporation of PHMG into bacterial cellulose (BC) films causes modifications of the IR spectra compared to the spectrum of pure BC. These changes were attributed to conformational changes that resulted from the formation of hydrogen bonds between the Cl^−^ ions of PHMG and the -OH groups of BC. However, we did not observe similar changes. A lack of a new vibration bands that could be attributed to the presence of PHMG functional groups in PVA-1.0 and PVA:Ch-1.0 films may have likely been caused by the bands that overlapped those of chitosan and PVA, as well as by a low (1 wt.%) content of PHMG. Due to the structural similarity of Ch and BC, it could be assumed that PHMG was bound to polymer matrix as a result of hydrogen interactions.

### 3.3. Atomic Force Microscopy

The application of different polymeric films in the industry (as a packaging material) or in medicine (as a wound dressing material and implants) is strongly dependent on biological fouling. Biofouling is regarded as an adhesion of different biological substances, like microorganisms, plants or animals (i.e., bacteria) to the analyzed surface, thus leading to the deterioration of surface properties or to damage [41]. The first step of biofilm formation is the adhesion of bacteria. Thus, a lot of effort in the modification of the polymeric films’ surface is devoted preventing bacteria from attaching themselves to the material, which subsequently hinders the overall rate and extent of biofilm formation.

Polymeric films that are classified as having antibacterial surfaces can be divided into two types: anti-adhesion surfaces and bactericidal surfaces [41,42]. The first type of surface is characterized by an unfavorable topography, impeding the process of bacterial cells affixing themselves to the material’s outer layer [43]. The bactericidal characteristic of the second type of surface is attributed to the presence of special additives or structures that cause the bacteria cell membrane to disintegrate.

**Figure 5:**
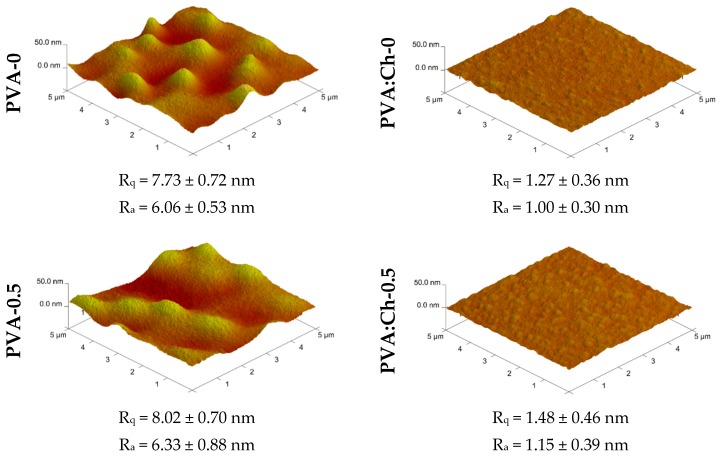

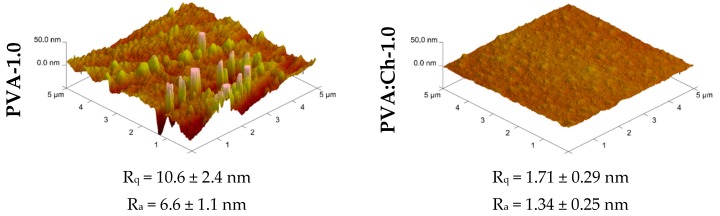
Atomic force microscopy (AFM) images of PVA- and PVA:Ch-based films.

It has been found that, in general, a higher surface roughness increases the adhesion of bacteria cells [44,45,46]. On the contrary, Liu et al. [47] for Ti-templated polydimethylsiloxane (PDMS), Lüdecke et al. [48] for titanium thin films, and Rizzello [49] for highly controlled nanorough gold surfaces suggested that a higher surface roughness does not influence bacteria adhesion and, in some cases, it even hinders this process. Even if the data presented in the literature are inconclusive, the effect of the modification on surface roughness of PVA and PVA:Ch films was studied. In Figure 5, AFM surface 3D images and the mean R_q_ and R_a_ parameters are presented.

It can be seen that both the one-component and two-component polymeric films exhibited defects like air bubbles, cracks, pores on nanoscale level.

There was also an evident correlation between surface structure, roughness parameters and PHMG content. For both types of films, increasing the PHMG content resulted in higher R_q_ and R_a_ parameters. The difference in R_q_ between the native and 1 wt.% PHMG-containing films was found to be 2.87 and 0.44 nm for PVA and PVA:Ch surfaces, respectively. Similar observations regarding the correlation between PHMG content and RRMS (root-mean-square) roughness were made by Nikkola et al. [50] for commercial thin-film-composite (TFC) polyamide (PA) membranes with PVA/PHMG coated surfaces.

It was clearly visible that the PVA:Ch films were substantially smoother than the corresponding PVA films. It is well known that the morphology of two-polymer mixtures is influenced by their mutual affinity and ability to blend [9,51,52]. As was found by other authors [53], in the case of hyaluronic acid films, the value of the roughness parameter and overall material roughness depends on the solvent used in order to prepare the films. Moreover, the differences in surface morphology are caused by the rise of ionic strength in the polymer solution, a rise which influences the decrease of electrostatic interactions between the polymer chains in the solvent. Generally, in the case of the polymeric blends, the final surface structure is not only affected by polymer/solvent interactions but also by polymer/polymer interactions, as well as the macromolecular structure. The molecular weight of both polymeric components and their fractions is also of great importance. The repulsive forces and/or electrostatic interactions between components in the blends may lead to an increase in the size of microdomains. Thus, the smoother surface of the Ch:PVA film was most likely the result of the intermolecular polymer interaction. Chitosan, being a cationic polyelectrolyte, forms a stretched chain in an acidic aqueous solution as a result of the electrostatic repulsion between protonated amino groups. Because PVA does not possess ionizable functional groups in its structure, the high compatibility between these two components—while mixing—was mainly the results of highly likely hydrogen bond formation. The H-bonding between PVA and Ch was already proven by FTIR analysis. These bonds prevented the aggregation of polymeric chains and improved mixing.

The difference in surface roughness observed in the case of the PVA-0 and PVA:Ch-0 films corresponded to the data presented earlier by Lewandowska et al. [54].

### 3.4. Differences in Color of PVA-Based Materials

It is well known that the color of packaging materials needs to fulfill customer expectations. For this reason, color seems to be of crucial importance for packaging manufacturers, affecting product appearance and shelf presence. We have found publications devoted to the psychology of color, where authors have convincingly suggested that emotional responses can be elicited by colors. Some of them are universal, while certain colors are characteristic of a country or even a particular region of culture and important in a particular context. Moreover, we have to bear in mind that colors can influence consumers in different, sometimes extreme ways, and, for this reason, the color selection process can be difficult. Producers employ various methods in order to determine and assess the attitude to a particular color of a product. One of these methods is the CIELab system, which was also applied in our work. The values of the measured parameters *L*, *a*, *b* and the calculated parameter Δ*E* are presented in Table 2.

Based on the obtained results, it could be seen that the amount of PHMG introduced into PVA did not significantly influence the changes in color of the obtained materials in comparison with pure PVA. In the case of the PVA-0.5 and PVA-1.0 samples, the values of the ΔE parameter were 0.4 and 0.3, respectively. It should be mentioned that visual changes in color have been found to be observed by common consumers when the ΔE value exceeds 2. The addition of chitosan to PVA led to a significant change in the color of the obtained material. Based on the data in Table 2, it was noted that some ΔE values exceeded 5, which meant that the colors of pure PVA and PVA:Ch-0 were clearly different. The most significant changes were observed in the case of parameter *b*, where color shifted to a more intensive yellow. In the case of samples consisting of PVA, chitosan and PHMG, the decrease in the *L* and *a* parameters was observed, while values of *b* significantly increased. This means that the studied materials tended to be darker with the increase in the PHMG content, and the color shifted to green–yellow. Moreover, it should be mentioned that ΔE parameter values in the case of materials based on PVA and chitosan mixture with a PHMG addition were 23.3 and 28.4, respectively. The obtained results confirmed that in the case of PVA-chitosan-based materials, the incorporation of PHMG will affect the appearance of products and, subsequently, the way consumers perceive products when PVA–Ch–PHMG films are applied.

### 3.5. Thermal Properties

**Figure 6:**
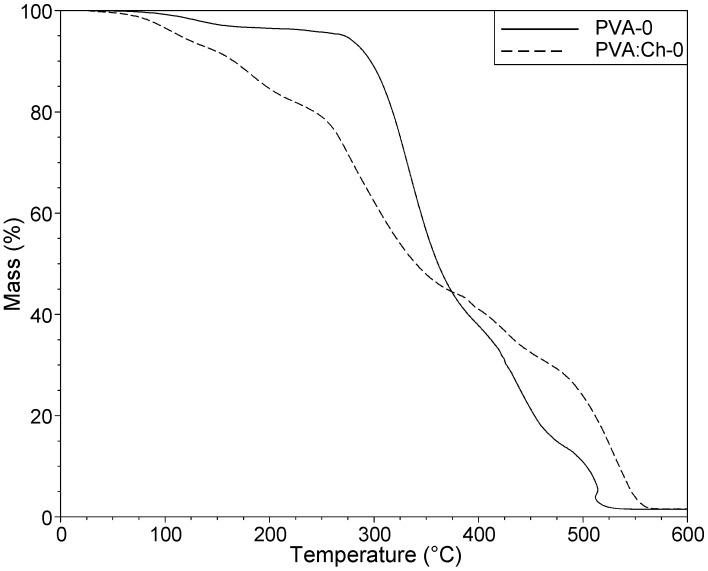
TG curves of the PVA-0 and PVA:Ch-0 samples.

In the case of polymers that can be used as packaging materials, thermal stability seems to be crucial. In order to determine the thermal stability of PVA-based materials, a TG analysis was performed. It is well known that the blending of polymers as well as the introduction of some additives can affect the thermal properties of obtained materials. For this reason, the impact of chitosan (Ch) and PHMG, acting as biocidal agents, on the thermal stability of PVA was established. The temperatures at 5%, 10% and 50% mass loss (T_5%_, T_10%_ and T_50%_) were selected as reference indexes (Table 3). After blending PVA with chitosan at a mass ratio of 50:50, the stability of the obtained PVA:Ch-0 sample decreased in comparison to the pure PVA film (Figure 6).

**Figure 7:**
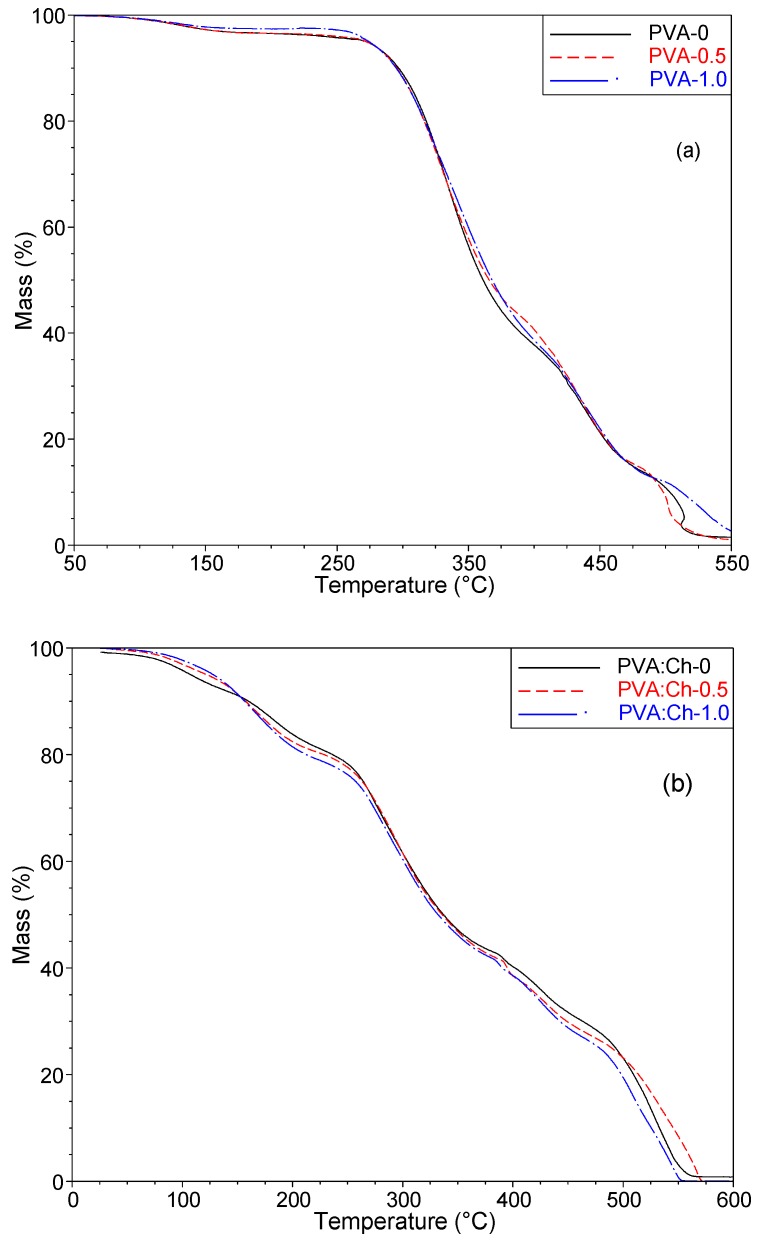
TG curves (**a**) of neat PVA-0 and PVA with different amounts of PHMG, as well as (**b**) neat PVA:Ch-0 and PVA:Ch with the addition of PHMG.

In the case of the PVA-0 samples, the first stage of decomposition was associated with moisture vaporization (80–150 °C). According to the literature [55,56], the second stage is connected with dehydration related to the formation of volatile products. In the last stage (at about 449 °C), further degradation occurs where carbon and hydrocarbons are produced. Taking the thermal properties of PVA and chitosan into account, it is well known that poly(vinyl alcohol) is more stable than chitosan [57,58]. For this reason, in the case of the PVA:Ch-0 material, the mass of the sample gradually decreased from the beginning of the heating process. The extent of the reduction in sample mass over time varied at particular stages of the heating process. After exceeding the temperature of 200 °C, a significant decrease of mass was observed, a decrease which may have been related to the glass transition of chitosan that, according to the literature [59], occurs at 211 °C. Moreover, on the TG curve of the PVA:Ch-0 sample, two ranges of temperatures (250–350 °C and 425–475 °C) could be noticed where the loss mass was more significant in comparison to pure PVA. The first one was due to the thermal degradation of both polymers that were part of the PVA:Ch-0 film, and this range indicated that the partial breaking of molecular structures and the disintegration of intermolecular bonds occurred. Based on the results presented in Figure 7, it can be observed that an introduction of PHMG into PVA and PVA-Ch-0 samples improved the stability of the analyzed materials during the first stage of degradation.

In the case of the PVA-PHMG systems, a slight increase of thermal stability in the initial phase of thermal treatment could be seen; however, in relation to the samples consisting of PVA, chitosan and PHMG, the changes in the T_5%_ values were more significant (Table 3). It should be mentioned that a shift to a higher temperature increased with an increased amount of PHMG in the studied materials. This observation suggests that the composition of PHMG, where in each individual mer three atoms of nitrogen were present, strongly influenced the thermal stability of the obtained materials. However, a higher stability was observed only for the temperature of the 5% mass loss. In the case of T_10%_ and T_50%_, the dependence mentioned above was not observed.

Based on the obtained data, it was concluded that the modification of the obtained polymer films with the biocidal agent, in the form of PHMG, affected their thermal properties. In particular, this modification increased their resistance to high temperatures in the initial range of measurements. These changes were minor in the case of PVA-based films; however, the study of films consisting of PVA, chitosan and the addition of PHMG indicated that the increase in T_5%_ was significant [60].

### 3.6. Mechanical Testing

**Figure 8:**
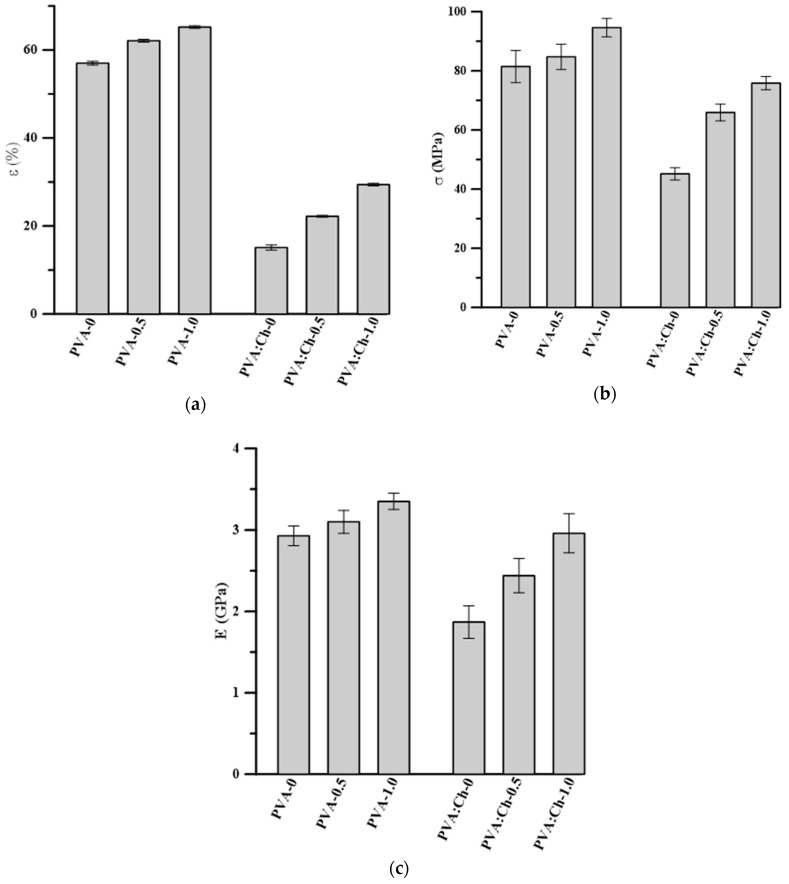
Comparison of (**a**) elongation at break; (**b**) tensile stress; and (**c**) Young’s modulus values of all studied polymeric films.

In the case of packaging materials, mechanical properties are crucial. A good packaging material is characterized by an adequate flexibility and tensile strength. For this reason, the elongation at break, tensile stress, and the Young’s modulus of the obtained materials were determined and are presented in Figure 8.

Taking into account poly(vinyl alcohol) films with and without PHMG, it could be clearly seen that the addition of the antibacterial compound increased the values of the Young’s modulus and tensile stress. Moreover, samples with PHMG were also more elastic than neat PVA. Considering the structure of PHMG, it can be assumed that hydrogen bonding influenced mechanical properties of the obtained materials. Moreover, PHMG is a viscous, low molecular weight substance, and, for this reason, the mechanical properties and the elongation at break of the obtained materials could have been improved due to the PHMG acting as a plasticizer.

The mixture of poly(vinyl alcohol) with chitosan at a mass ratio of 50:50 (PVA:Ch-0) was characterized by lower values of mechanical properties in comparison with pure PVA. This is in accordance with the literature, where materials based on poly(vinyl alcohol) and chitosan have been described [55,56,61]. As described by other authors [55,56,61], the tensile strength of chitosan and PVA–chitosan blends is significantly lower than in the case of pure PVA. The results of mechanical testing obtained by us and other researchers strongly indicate lower elongation at break, tensile stress, and Young’s modulus values of neat chitosan, in comparison to PVA. The introduction of PHMG into a PVA–Ch matrix significantly influences the latter’s mechanical parameters. As was mentioned above, in the case of PVA, the addition of poly(hexamethylene guanidine) increased all discussed mechanical properties; however, the changes in relation to the PVA and chitosan mixture were even more significant. This suggests that the improvement of mechanical properties was due to the interaction between the polymer, acting as the matrix, and PHMG hydrogen bonds [4]. The highest values of all studied mechanical parameters—the elongation at break, tensile stress, and Young’s modulus—were observed in the case of the PVA:-Ch-1.0 sample, which seemed to be the best packaging material among all of the studied samples. The same observation was made by Chen at al. [4], who indicated that the H-bond network after the addition of 1.0 wt.% PHMG seemed to be at an optimized state.

### 3.7. Surface Free Energy Analysis

It is well known that bacterial adhesion is initiated by electrostatic attraction, hydrophobic interactions, and Van der Waals forces between the surface of bacteria cells and the surface of the colonized material. Moreover, in the work of Kocijan et al. [62], it was shown that surface topography and surface energy are key factors in the adhesion between materials and the cells of microorganisms. The surface morphology of analyzed materials was described in the section devoted to the AFM technique. The second of the above-mentioned factors that can influence the cell adhesion is surface free energy (SFE). Measuring the contact angle is a method which allows for the assessment of SFE and its particular components: dispersive and polar. This technique also enables the determination of the hydrophilicity or hydrophobicity of the material surface and thus helps to assess the biocidal potential of the material surface in contact with the biological environment. In Table 4, the values of surface free energy and its polar and dispersive components are presented. It is evident that in the case of the sample consisting of PVA and chitosan (PVA:Ch-0), the value of surface free energy was significantly lower than in that of PVA. The same tendency was observed in the publication of Lewandowska [63]. The obtained results suggest that in the mixture of PVA and chitosan, the polar groups present in the chains of the polymers used in the study could be diverted towards the inner layer of the studied film. The introduction of PHMG into PVA as well as into PVA–chitosan system caused an increase in the surface free energy value. This is consistent with the results described in work of Moshynets et al. [60], where the authors indicated that the addition of PHMG enhanced the hydrophilicity of a newly obtained material.

The mechanism of bacterial adhesion to the biomaterial surface is very complicated; however, most publications have indicated that irregularities of polymer surface increase bacterial adhesion and biofilm deposition [64]. In the case of surface free energy, we took both the hydrophobicity of polymeric surface and the strain of bacteria into account. The majority of research has suggested that hydrophilic materials are more resistant to bacterial adhesion than the hydrophobic ones [65]. In order to comprehensively discuss the SFE values, both polar and dispersive components have to be taken into account. In the present work, the obtained results indicate that, in the case of all studied materials, the dispersive component values were much higher than the polar component. This justifies the assumption that the PVA-based materials without and with the addition of PHMG had a primarily hydrophobic surface. According to the literature, *E. coli* and *S. aureus* belong to the group of non-halophilic bacteria [66]. For this reason, they display hydrophobic properties and most readily attach themselves to hydrophobic materials. However, Chen et al. [67] indicated that bacteria can display an affinity to hydrophilic surfaces with a positive or neutral charges. Taking the obtained results and information accessible in literature into account, it is reasonable to assume that roughness and surface free energy play an important role in the antibacterial properties of PVA-based materials. However, we have to bear in mind that the effect PHMG has on microorganisms is a result of the presence of positive charges that are able to compensate for the negative charges present on the cell walls of bacteria.

### 3.8. Assessment of Antibacterial Potential

In order to evaluate the antibacterial properties of PVA-based materials, analyses were performed by means of two methods: The first one was the Kirby–Bauer (disk diffusion) method, and the second one was the dilution and pour plate culture method. Initially, the antibacterial potential of PVA materials was determined by the diffusion technique based on the analysis of bacteria-free areas around the samples. The results obtained by means of the above-mentioned methods are presented in Table 5.

A comparison of antibacterial properties of pure PVA, the PVA:Ch-0 blend, and the PVA and PVA:Ch samples with the addition of PHMG was performed by using the visualization method. As expected, a pure PVA film did not exhibit an antibacterial capacity. In the case of the PVA:Ch-0 sample, compared to the PVA film, a reduction in the proliferation intensity of *S. aureus* and *E. coli* in direct contact between the material and the inoculated agar was observed. However, around the PVA:Ch-0 film, no inhibition zone was observed. It is reasonable to assume that the lack of an inhibition zone around the PVA:Ch-0 sample resulted from the entrapment of active, positively-charged chitosan molecules between PVA chains. This phenomenon is probably related to the comparatively large size of biopolymer molecules and the strong hydrogen bonds formed between chitosan and PVA [1,2]. Moreover, as the pH of agar is close to 7.4, in the studied conditions, only residual −NH_3_^+^ functional groups were present in the PVA:Ch-0 polymer matrix. As described by Qin et al. [19], the lack of protonated amino groups in chitosan also decreases its antibacterial properties. However, the results obtained by the means of the disk diffusion method indicated that antibacterial capacity of the samples containing Ch was higher than that of neat PVA.

In the case of the materials based on PVA and PVA:Ch blends containing PHMG, inhibition zones of microorganisms were observed (Table 5). Moreover, significantly larger bacteria-free areas for the PVA film that contained PHMG, in comparison to the ternary composites, were noted. Based on previously described results of FTIR and AFM analyses, it was established that this phenomenon resulted from the significant extent of interactions between individual components of the studied materials. Hydrogen bonds formed between chitosan and poly(vinyl alcohol) contributed to the decrease in the biologically active surface of the composites that contained chitosan. Moreover, the interactions of the functional groups of Ch and PVA most likely formed a dense network and significantly reduced the migration of PHMG to the outer layers of the composites.

In order to confirm antibacterial activity of the obtained polymer films, as well as to quantify their capacity to combat bacteria, the dilution and pour plate culture method was applied. The numbers of bacterial colonies for individual samples and the values of antibacterial potential of materials are listed in Table 6.

Similarly to the Kirby–Bauer method, the proliferation of both *S. aureus* and *E. coli* in an agar medium with an immersed neat PVA film was observed. In the case of the PVA:Ch-0 film, the antibacterial effect of the material affected and limited only half of the colony-forming units of bacteria. Taking into account the obtained results, it should be mentioned that in the works of Liu [6] and Benhabiles [3], the antimicrobial activity of films containing chitosan was found to be related to selected features of a biopolymer, namely M_w_ of polysaccharide, the duration of the biocidal test, and the stage of *E. coli* proliferation at which the sample was immersed in the microorganism suspension. For this reason, it is reasonable to assume that the low capacity of the PVA:Ch-0 film consisting of 50 wt.% of chitosan to inhibit the proliferation of Gram-negative bacteria could have also been related to the above-mentioned factors. Similarly, the low antimicrobial properties of the Ch-containing films established in the disc diffusion method could be related to the basic pH of the applied nutrient broth. According to Qin et al. [19], in non-acidic conditions, chitosan can act as nutrient for bacteria growth.

In summary, the strongest antimicrobial effect was observed in the case of the samples consisting of PVA and PHMG, regardless of the amount of the additive. These films, according to the results obtained by Chen [4], were characterized by means of the capacity to completely inhibit bacteria growth. In this context, the addition of chitosan improved the bactericidal properties of the neat PVA films. In turn, chitosan hindered these properties when PHMG was incorporated and the synergistic effect was not observed.

## 4. Conclusions

Novel bioactive materials based on poly(vinyl alcohol), chitosan and PHMG were successfully obtained, and their structure was determined by means of the NMR and FTIR techniques. It was found that all used components were compatible and interacted with each other through hydrogen bonds. The analysis of these materials’ mechanical properties proved that PHMG acted as plasticizer and increased the elasticity of the obtained materials. The same tendency was observed in the case of bactericidal properties, especially in relation to films consisting of PVA and PHMG. Moreover, the introduction of poly(hexamethylene guanidine) into poly(vinyl alcohol), as well as into the PVA-chitosan mixture, significantly benefited the thermal stability of the studied materials. This phenomenon is related to the presence of nitrogen atoms in the structure of PHMG that hindered the diffusion of oxygen into polymer matrixes. An evaluation of the color changes of the obtained materials showed that PHMG played an important role by acting as a dye. Research into the changes in color indicated that the introduction of PHMG into PVA does not influence the color observed by a prospective consumer, while materials consisting of PVA, chitosan, and PHMG were characterized by an increase of the ΔE parameter that was significantly higher than two. Moreover, it should be noted that the results of the AFM study demonstrated that the addition of poly(hexamethylene guanidine) did not influence the surface of the obtained films. Due to their strong hydrogen bonding, PVA:Ch films were substantially smoother than the corresponding PVA ones. A contact angle analysis confirmed the reduction in surface free energy after the incorporation of chitosan into the PVA matrix, contrary to an effect of PHMG. Thus, PHMG was found to enhance the hydrophilicity of the obtained materials, resulting the obtainment of a surface that was more resistant to bacterial adhesion.

Interestingly, the performed analyses allowed us to establish that materials based on PVA and PHMG were characterized by more significant antibacterial properties than the films consisting of a PVA-chitosan system with the addition of PHMG. This indicates that the simultaneous introduction of both biologically active compounds chitosan and PHMG did not synergistically improve the antimicrobial properties of the PVA-based materials.

To summarize, based on the obtained results, it has been established that the formed films are interesting products that can be considered as potential materials in packaging with improved antibacterial properties.

## Figures and Tables

**Table 1:** Polymeric films’ compositions.

Sample	PVA:Ch Mass Ratio	PHMG Content wt.%
PVA-0	100:0	0.0
PVA-0.5		0.5
PVA-1.0		1.0
PVA:Ch-0	50:50	0.0
PVA:Ch-0.5		0.5
PVA:Ch-1.0		1.0

**Table 2:** Color variables.

Sample	Color Variables			
	*L*	*a*	*b*	Δ*E*
PVA-0	92.4	1.2	−11.6	−
PVA-0.5	92.0	1.2	−11.6	0.4
PVA-1.0	92.7	1.2	−11.7	0.3
PVA:Ch-0	90.9	−1.8	5.1	17.0
PVA:Ch-0.5	90.0	−2.9	11.2	23.3
PVA:Ch-1.0	89.4	−3.5	16.2	28.4

**Table 3:** Thermogravimetric (TG) data for all studied materials.

Sample	T_5%_ [°C]	T_10%_ [°C]	T_50%_ [°C]
PVA-0	272.0	296.8	361.3
PVA-0.5	272.1	295.5	365.9
PVA-1.0	274.1	295.0	368.1
PVA:Ch-0	112.6	165.4	340.1
PVA:Ch-0.5	122.0	157.3	335.8
PVA:Ch-1.0	127.9	156.6	332.0

**Table 4:** Surface free energy ($\gamma_{S}$) and its dispersive ($\gamma_{S}^{d}$) and polar ($\gamma_{S}^{p}$) components for PVA-based materials.

Sample	$\gamma_{S}^{d}\;[\mathrm{mJ}/m^{2}]$	$\gamma_{S}^{p}\;[\mathrm{mJ}/m^{2}]$	$\gamma_{S}\;[\mathrm{mJ}/m^{2}]$
PVA-0	34.67 ± 0.85	4.43 ± 0.27	39.10 ± 1.12
PVA-0.5	35.27 ± 0.17	4.77 ± 0.23	40.04 ± 0.39
PVA-1.0	37.10 ± 0.20	3.95 ± 0.16	41.05 ± 0.35
PVA:Ch-0	34.67 ± 0.21	2.41 ± 0.11	37.07 ± 0.32
PVA:Ch-0.5	35.11 ± 0.16	2.91 ± 0.18	38.02 ± 0.15
PVA:Ch-1.0	35.90 ± 0.10	3.00 ± 0.22	38.90 ± 0.21

**Table 5:** Inhibition zones against different bacteria exhibited by all studied films.

Sample	Width of the Inhibition Zone [mm]	
	*S. aureus*	*E. coli*
PVA-0	0 ^a^	0 ^a^
PVA-0.5	4	2
PVA-1.0	3–4	3–4
PVA:Ch-0	0 ^b^	0 ^b^
PVA:Ch-0.5	1–2	1
PVA:Ch-1.0	1	1

^a^ Growth of bacteria throughout the agar and under the specimen; ^b^ growth of bacteria under the specimen partially inhibited.

**Table 6:** The number of viable bacteria recovered in 1 mL (N) and the antibacterial activity (R) of the PVA-based material.

Sample	*S. aureus*		*E. coli*	
	N (CFU/mL)	R	N (CFU/mL)	R
PVA-0 (blank)	3.7 × 10^5^	5.6 ($log\;N_{blank})$	1.0 × 10^6^	6.0 ($log\;N_{blank})$
PVA-0.5	<1	≥5.6	<1	≥6.0
PVA-1.0	<1	≥5.6	<1	≥6.0
PVA:Ch-0	2.9 × 10^3^	2.1	2.8 × 10^5^	0.56
PVA:Ch-0.5	2.9 × 10^3^	2.1	5.9 × 10^4^	1.2
PVA:Ch-1.0	3.7 × 10^3^	2.0	2.6 × 10^4^	1.6

## Supplementary Materials

The following are available online at https://www.mdpi.com/2073-4360/11/12/2093/s1, Figure S1: FTIR-ATR spectra of PVA-0 and PVA-1.0, PVA:Ch-0 and PVA:Ch-1.0.
