# Supplementary material for: Antibacterial Films Based on PVA and PVA–Chitosan Modified with Poly(Hexamethylene Guanidine)
Source: Polymers (Basel). 2019 Dec 13;11(12):2093. doi: 10.3390/polym11122093 (PMC6960635; doi:10.3390/polym11122093)
Supplement: Supplementary file 1 [file polymers-11-02093-s001.pdf]

polymers

Supplementary Materials

Antibacterial Films Based on PVA and PVA–Chitosan Modified with Poly(Hexamethylene Guanidine)

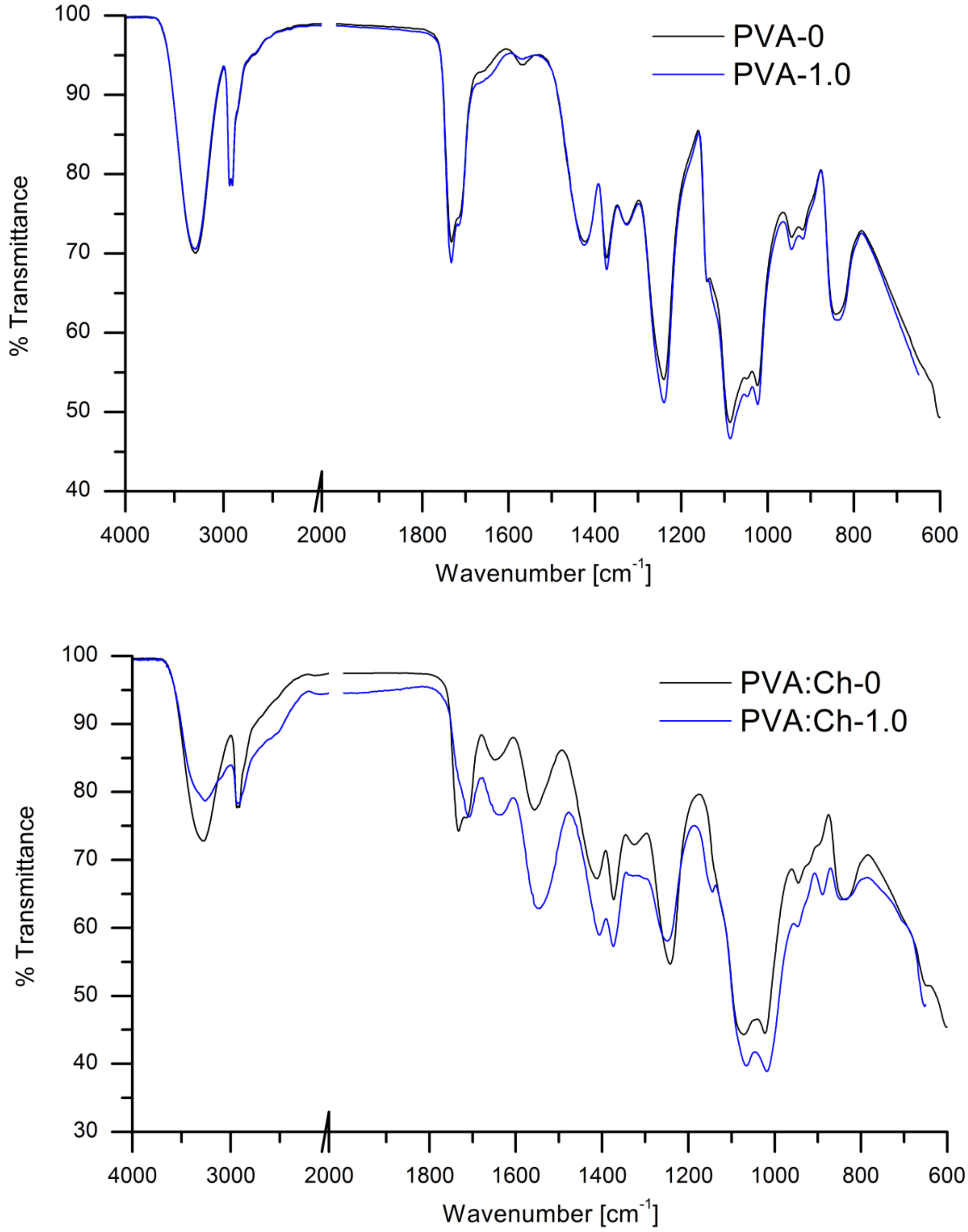

Figure S1. FTIR-ATR (Fourier transform infrared–attenuated total reflectance) spectra of PVA-0 and PVA-1.0, PVA:Ch-0 and PVA:Ch-1.0.
